# Non-Melanoma Skin Cancer Mortality in Spain: A Predictive Model up to 2044

**DOI:** 10.3390/jcm10245750

**Published:** 2021-12-08

**Authors:** Mercedes Sendín-Martin, Juan Carlos Hernández-Rodríguez, Antonio-José Durán-Romero, Juan Ortiz-Álvarez, Julian Conejo-Mir, José-Juan Pereyra-Rodríguez

**Affiliations:** 1 Dermatology Departement, Hospital Universitorio Virgen del Rocio, Avda. Manuel Siurot s/n., 41013 Sevilla, Spain; mariam.sendin.sspa@juntadeandalucia.es (M.S.-M.); juanc.hernandez.sspa@juntadeandalucia.es (J.C.H.-R.); antonioj.duran.sspa@juntadeandalucia.es (A.-J.D.-R.); juan.ortiz.alvarez.sspa@juntadeandalucia.es (J.O.-Á.); jconejomir@us.es (J.C.-M.); 2 Medicine Department, Universidad de Sevilla, 41004 Sevilla, Spain

**Keywords:** non-melanoma, skin cancer, epidemiology

## Abstract

Non-melanoma skin cancers (NMSC) are the most common malignancies worldwide and are, worryingly, increasing in incidence. However, data in the literature on NMSC specific mortality are scarce, because these tumors are excluded from most mortality registries. The main objective of this study is to analyze NMSC’s mortality rates and use them to generate a predictive model for the coming years in Spain. Data on mid-year population and death certificates for the period 1979–2019 were obtained from the Spanish National Statistics Institute. The Nordpred program (Cancer Registry of Norway, Oslo, Norway) within statistical program R was used to calculate mortality adjusted rates, as well as the mortality projection with an age-period-cohort model. This is the first study to report a prediction about NMSC mortality in the next years. According to our findings, the number of NMSC deaths in older people will grow in both sexes, especially in those older than >85 years old (y.o.). The age-specific mortality rates of NMSC will tend to stabilize or gradually decrease, with the exception of women between 75–79 y.o., who will present a slight increase at the end of the period. Early prevention and screening of NMSC specifically oriented to this population might change this tendency.

## 1. Introduction

Skin cancer has traditionally been divided into melanoma and non-melanoma skin cancers (NMSC). Non-melanoma skin cancers consist of the most common tumors worldwide and their prevalence is higher than the sum of all other cancers combined [1]. This group of tumors includes common malignancies such as basal cell carcinoma (BCC) and squamous cell carcinoma (SCC), but also rare tumors such as Merkel cell carcinoma or atypical fibroxanthoma. Traditionally, mortality in this NMSC group has been considered low, especially in the case of BCC. However, mortality from SCC has increased significantly in recent years, especially in immunocompromised or elderly patients [2].

Currently, mortality due to SCC appears to be underreported [2] and it’s considered an increasing worldwide problem. There are recent studies reporting that SCC related mortality in high-incidence areas may be equivalent to mortality associated with other tumors, such as renal or oropharyngeal carcinomas and melanoma [3]. Nevertheless, data in the literature on SCC-specific mortality are scarce, because this group of tumors is excluded from many mortality registries [4]. Most national mortality registries include SCC mortality under the heading of NMSC, which also includes other entities mentioned above, such as BCC or Merkel cell carcinomas. However, given the extremely low mortality of BCC (near to none) [1,2,5] and the low incidence of other skin tumors, such as Merkel cell or adnexal tumors [6,7,8,9], the majority of deaths attributed to NMSC would correspond to SCC, as has been previously pointed out in the literature [4,10].

Annual mortality from SCC in the US has been estimated at 2500 and 8000 cases per year in two different studies [3,10]. Concerning Spain, annual incidence of SCC has been calculated at 38.16 per 100,000 person-years [4], but there are scarce data about mortality. Only one recent study analyzes the mortality associated with NMSC in Spain in the last few years [11]. To the best of our knowledge, there is no study that predicts mortality trends for NMSC in Spain in the next few years. The main objective of this study is to analyze NMSC mortality rates in Spain and use them to generate a predictive model for the next few years up to 2044.

## 2. Materials and Methods

Data on mid-year population and death certificates for the period 1980–2019 were obtained from the Spanish National Statistics Institute (NSI) (http://www.ine.es, accessed on 26 July 2021). The population data for the 2020–2044 period were obtained from the official population projection figures provided by the NSI. All death records, obtained in microdata files, which included NMSC (International Classification of Diseases (ICD): ICD-9-CM: 173; ICD-10: C-44) as the cause of the death were included in the study. Age-specific mortality rates by 5-year age groups were computed.

**Figure 1:**
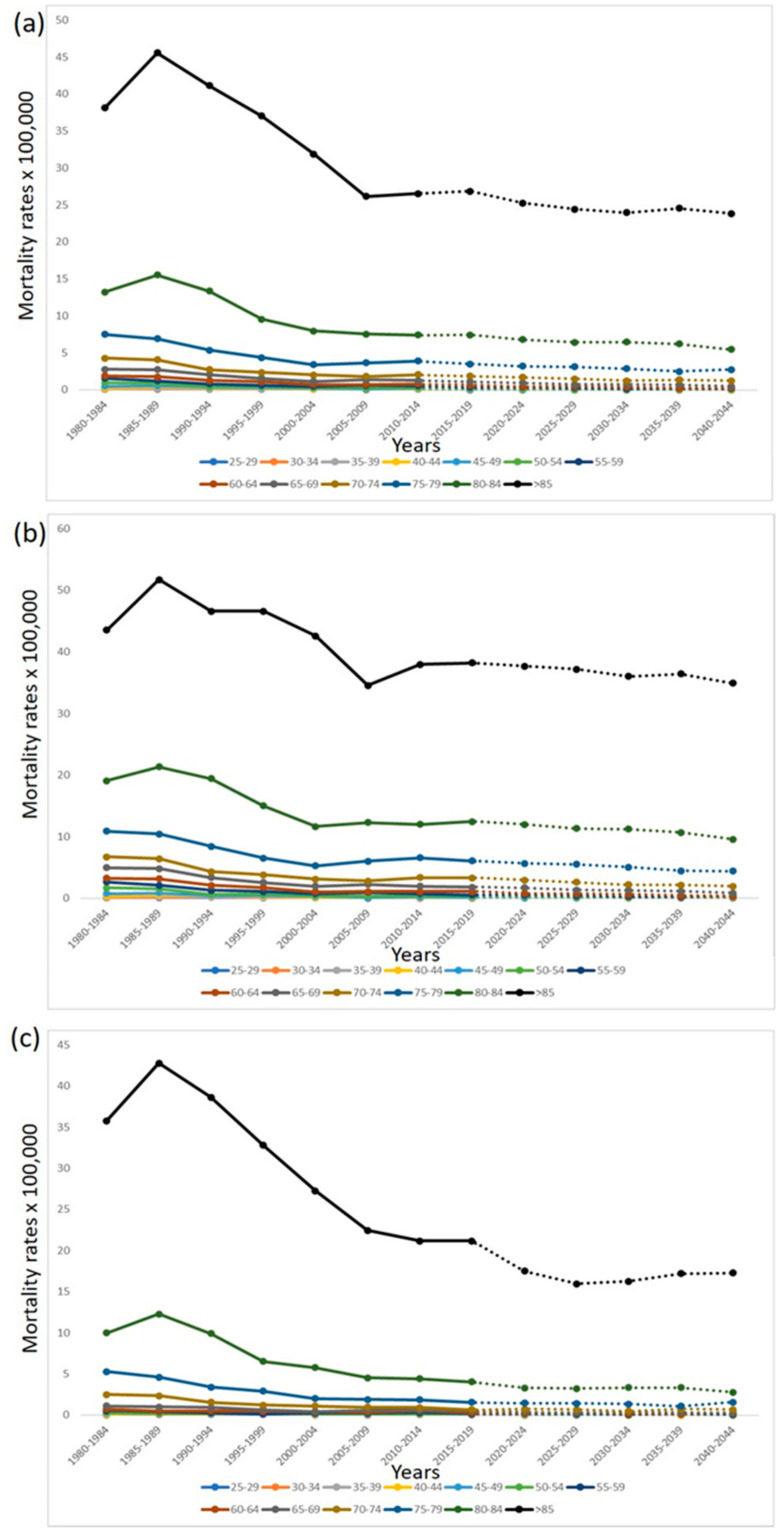
Observed (1980–2019) and predicted (2020–2044) age-specific mortality rates for NMSC in Spain. (**a**) Both sexes; (**b**) males; (**c**) females.

The Nordpred program (Cancer Registry of Norway, Oslo, Norway) [12] within statistical program R [13] was used to calculate age-specific mortality rates, as well as the mortality projection. The code in R project is provided as Supplementary Material. The European standard population 2013 was used for standardization. We used the R package Nordpred to calculate an age-period-cohort model. Data were compiled in five-year blocks. We extrapolated the most recent 10-year linear trend, attenuating the slope by 25% and 50% in the second and third prediction periods, and by 75% in the fourth and fifth periods, as recommended by the authors following empirical validation [12]. The results of the predictions are presented for the total of observed and expected deaths for each period in Spain. For each period, adjusted mortality rates were calculated based on the age-standardized rates. The annual changes in the number of deaths were calculated for the last projected period (2040–2044) in comparison with the last observed period (2015–2019), where the proportion of this change could be due to changes of risk of death due to NMSC or due to demographic changes (size or structure of population). These two components could be different from zero and present a positive or negative direction. According to our data, the population is not ageing during the study time, so we attribute our results to real changes in mortality trends. Nordpred uses the Power5 and Poisson age-period-cohort models to calculate prediction of mortality from 2020 to 2044 [14]. Since statistical methods based on age-period-cohort models often overestimated the number of cases due to their exponential growth over time, we decided to use Power5 function to level this off and improve our main predictions (Table 1 and Table 2 and Figure 1). However, a comparison between Poisson and Power5 predictive models is provided at the end of the text.

Finally, GraphPad Prism version 6.0 (GraphPad Software, San Diego, CA, USA) was used for graphics of mortality trends.

## 3. Results

In the period 1980–2019, there were 20,965 deaths from NMSC in Spain. By sex, there were 11,792 NMSC deaths in males and 9173 in females. The predictive model applied predicts that in the period 2020–2044 there will be 18,918 deaths from NMSC in Spain in both sexes, with the age group > 85 years old (y.o.) and males showing the highest number of deaths. The detail of the number of deaths produced in Spain (1980–2019) and its prediction (2020–2044), according to age groups and sex, can be seen in Table 1.

The standardized mortality rate ranged from a minimum of 1.25 deaths per 100,000 people in the period 2015–2019, to a maximum of 2.5 deaths per 100,000 inhabitants in the period 1985–1989. Sex-specific mortality rates ranged from 0.54 to 1.16 deaths per 100,000 inhabitants in males versus 0.21–0.5 in females. The predictive model up to 2044 predicts mortality rates that range from 0.25 deaths per 100,000 inhabitants in the period 2030–2034 and 0.31 in 2020–2024. By sex, mortality rates in men were estimated between 0.38 in 2040–2044 and 0.49 in the period 2020–2024. Regarding women, 0.16 in 2040–2044 and 0.18 deaths per 100,000 inhabitants in the 2020–2024 period were expected. Table 2 shows the standardized mortality rates for the observed and predicted periods.

**Figure 2:**
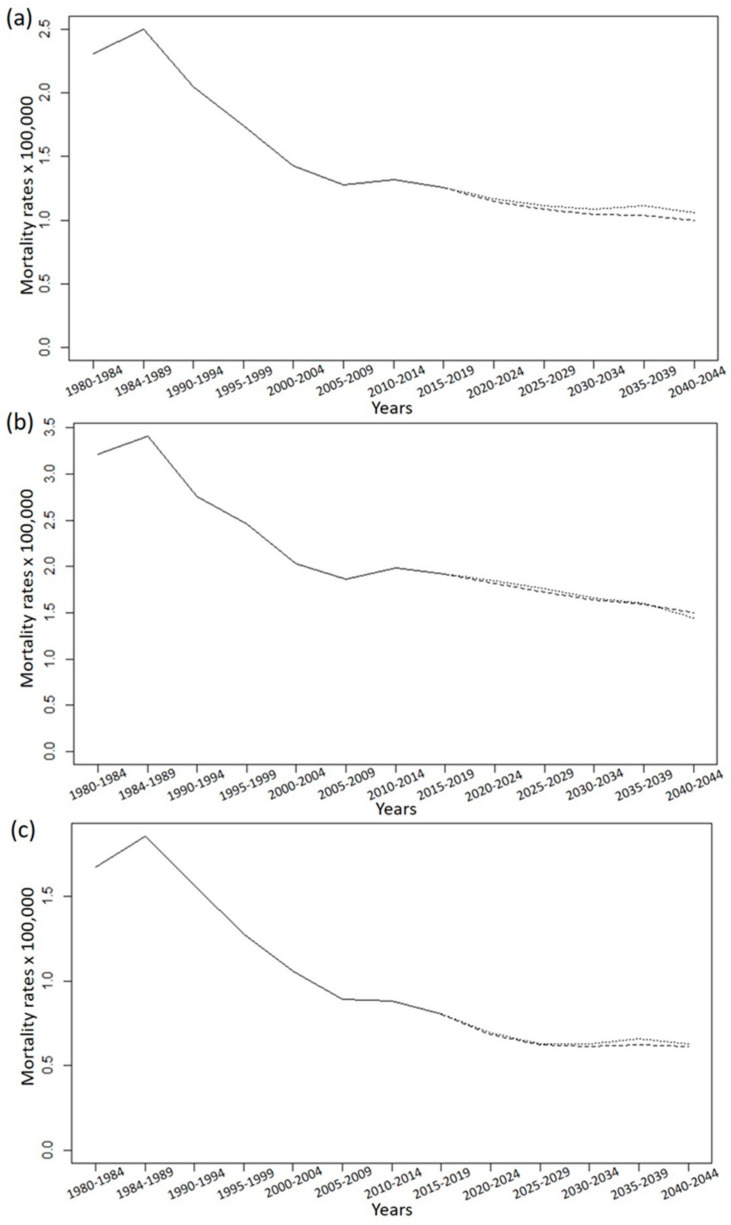
Observed (1980–2019) and predicted (2020–2044) overall mortality rates for NMSC in Spain. (**a**) Both sexes; (**b**) males; (**c**) females. Dashed line: Prediction Power5. Dotted line: Poisson.

Figure 1 shows the age-specific mortality rates for the observed and predicted periods for both sexes. In most age groups, the predictive model tends to stabilization or decline of NMSC mortality, with the exception of women between 75 and 79 years old (y.o.). In this latter group an increase in mortality at the end of the period is expected (see Figure 1c). The same trend can be seen in Figure 2, which shows the overall mortality rate in the periods studied (observed and predicted).

## 4. Discussion

The present study reports for the first time a prediction of NMSC mortality for the coming years in Spain. According to our findings, the number of NMSC deaths in older people will grow in both sexes, especially in those older than >85 y.o. In the younger age groups, the number of deaths will remain mostly stabilized in compliance with our prediction model. In general, the age-specific mortality rates of NMSC will tend to stabilize or gradually decrease, with the exception of women between 75 and 79 y.o., who will present a slight increase at the end of the period.

Nowadays, mortality associated with NMSC is a growing problem, justifying even the use of the term “skin cancer epidemic” [15]. This situation is further accentuated by the fact that precise data about NMSC are not available. Many cancer registries do not include NMSC or record only the first tumor per patient [4,16]. For example, new cases of BCC and SCC in the US are not included in national cancer registries and are excluded from the National Cancer Institute’s Surveillance, Epidemiology and End Results (SEER) [17] program, in contrast to other malignant cancers that are indeed included. In Spain, data on NMSC incidence and mortality are included in the annual registry of the National Centre for Epidemiology (Carlos III Health Institute) [18] and the Spanish National Statistics Institute. However, data regarding NMSC are not usually stratified by tumor subtype, so all skin cancers other than melanoma are included within this single category. As a consequence, there are no reliable or precise epidemiological data available for each separate tumor type.

In the future, improving NMSC data collection is essential to avoid underestimation of its incidence and mortality. Up until this point, almost all mortality in the NMSC group could be reasonably attributed to SCC, as has already been reported in previous studies [4,10]. In comparison to BCC and SCC, the rest of tumors within NMSC group (such as Merkel cell carcinoma, dermatofibrosarcoma protuberans, or primary cutaneous lymphoma) are remarkably infrequent [7,19,20]. On the other hand, BCC presents high incidence, but the mortality associated with it is practically none [5,21]. SCC is the second most common tumor from NMSC group, with an incidence that ranges from 5 to 499 per 100,000 patients depending on the latitude [22]. Although most SCCs are treated locally with no sequelae, SCCs associated with risk factors (diameter of at least 2 cm, poor differentiation, perineural invasion, etc.) present a significant risk of metastasis and death [23]. In view of the above arguments, NMSC mortality can be understood as mostly equivalent to SCC mortality.

To the best of our knowledge, this is the first study predicting mortality trends for NMSC in Spain. According to our results, the net number of deaths from NMSC in the elderly will grow in both sexes, especially in those >85 y.o. (Table 1). However, our model predicts that the standardized mortality rates of NMSC mortality will tend to stabilize or will gradually decrease (Table 2, Figure 1 and Figure 2). The most notable exception to this trend will be women between 75 and 79 y.o., who will present even a slight increase in their rates at the end of the period (2040–2044). A recent study from Germany predicted a significant increase of NMSC incidence over the next few years, whereas NMSC mortality will remain stable or even decrease [24]. This same tendency toward stabilization or decrease will be observed in Spain in the following years according to our results (Figure 1 and Figure 2).

NMSC mortality rates are strongly associated with sex and age. After disaggregating our results by sex and age, it is remarkable how women between 75 and 79 y.o. will increase their mortality rate at the end of the predicted period (Figure 1). Previous studies have shown that the highest NMSC mortality rates are reported in elderly males, although the global mortality trend in this group is to stabilize and decrease [25,26]. In contrast, a previous study observed the same concerning increase in relative risk of NMSC mortality in women born after the 1970s [11]. According to our prediction, this sex/age group will suffer an increase of NMSC mortality. This fact should be taken into consideration to design public policies for early prevention and screening of NMSC specifically oriented to this group.

Currently, most NMSC are treated with surgical removal. However, not all tumors are eligible for surgical intervention. Also, aggressive tumors, such as SCCs, can relapse, so radiotherapy and systemic immunotherapy and chemotherapy may be needed in the management of these tumors [27,28]. New PD-1 blockade drugs like cemiplimab are increasingly utilized for SCC [29,30], and promisingly they will decrease even more the mortality associated with SCC in the next few years.

Our study presents several limitations. Firstly, death certificates are frequently affected by errors that can affect subsequent mortality rates analysis [31]. Secondly, predictions of mortality are necessarily affected by changes in NMSC diagnosis or treatment, so they should be interpreted cautiously because NMSC might change in the coming years. In addition, as has been discussed, death-certificate information is neither homogeneous nor consistent between different countries. As a consequence, to improve our ability to predict mortality trends in the near future, it is desirable that death certificates become uniform between different countries. 

## 5. Conclusions

This is the first study to report a prediction about NMSC mortality in the next years in Spain. There are very few studies considering the tendencies in NMSC in the near future, but the stabilization or decrease of NMSC mortality rates is common within them. According to our findings, the age-specific mortality rates of NMSC will follow this tendency of stabilization. Our model predicts an increase in the number of deaths from NMSC in the elderly, especially in those older than 85 y.o. Prevention measures for early diagnosis of NMSC and public policies might need to be emphasized in this age group, because of the expected rise in NMSC mortality.

## Figures and Tables

**Table 1:** Number of deaths observed (1980–2019) and projected (2020–2044) by periods and age groups by NMSC in Spain. (**A**) Both sexes; (**B**) men; (**C**) women.

(**A**) **Both Sexes**													
**Observed NMSC Deaths**									**Projected NMSC Deaths**				
**Age** (**Years**)	**1980**–**1984**	**1985**–**1989**	**1990**–**1994**	**1995**–**1999**	**2000**–**2004**	**2005**–**2009**	**2010**–**2014**	**2015**–**2019**	**2020**– **2024**	**2025**–**2029**	**2030**–**2034**	**2035**–**2039**	**2040**–**2044**
**0**–**4**	0	1	0	0	0	0	0	0	0	0	0	0	0
**5**–**9**	1	1	1	0	0	1	0	0	0	0	0	0	0
**10**–**14**	1	0	1	0	0	0	0	0	0	0	0	0	0
**15**–**19**	3	3	3	0	1	1	0	0	0	0	0	0	0
**20**–**24**	2	4	1	2	5	1	1	2	2	2	2	2	2
**25**–**29**	5	13	13	13	7	3	7	2	4	4	4	5	4
**30**–**34**	11	16	21	21	8	6	8	5	3	2	2	2	2
**35**–**39**	16	30	18	18	13	12	10	7	5	4	3	3	3
**40**–**44**	24	43	32	29	16	20	29	9	10	7	6	5	5
**45**–**49**	55	51	38	38	30	17	37	21	12	12	10	8	7
**50**–**54**	113	98	41	56	37	35	49	43	34	20	20	18	15
**55**–**59**	163	138	86	60	56	76	76	58	64	53	34	37	33
**60**–**64**	162	176	135	114	69	82	100	90	74	86	76	53	58
**65**–**69**	200	216	193	156	117	129	140	135	127	113	136	127	93
**70**–**74**	265	258	196	201	188	172	182	195	195	186	176	218	207
**75**–**79**	333	348	283	264	247	303	333	279	302	321	323	324	397
**80**–**84**	330	483	484	375	365	433	498	524	446	508	562	590	608
**>85**	533	834	990	1128	1134	1146	1540	1914	2049	2057	2312	2724	3006
**Total number of deaths**	2217	2713	2536	2475	2293	2437	3010	3284	3212	3373	3663	4115	4440
(**B**) **Men**													
**Observed NMSC Deaths**									**Projected NMSC Deaths**				
**Age** (**Years**)	**1980**–**1984**	**1985**–**1989**	**1990**–**1994**	**1995**–**1999**	**2000**–**2004**	**2005**–**2009**	**2010**–**2014**	**2015**–**2019**	**2020**– **2024**	**2025**–**2029**	**2030**–**2034**	**2035**–**2039**	**2040**– **2044**
**0**–**4**	0	0	0	0	0	0	0	0	0	0	0	0	0
**5**–**9**	1	1	1	0	0	0	0	0	0	0	0	0	0
**10**–**14**	1	0	1	0	0	0	0	0	0	0	0	0	0
**15**–**19**	3	2	2	0	1	1	0	0	0	0	0	0	0
**20**–**24**	1	1	0	1	2	0	1	0	1	1	1	1	1
**25**–**29**	4	7	11	9	6	1	3	0	1	1	1	2	1
**30**–**34**	8	10	19	15	5	5	3	4	3	2	2	2	2
**35**–**39**	11	18	14	16	9	8	7	3	4	3	3	3	3
**40**–**44**	19	32	25	20	10	13	18	5	4	5	4	4	4
**45**–**49**	42	43	28	30	20	11	20	13	8	5	7	6	6
**50**–**54**	97	87	32	41	30	26	40	29	25	15	10	14	13
**55**–**59**	134	114	70	54	43	60	53	45	46	40	26	19	26
**60**–**64**	129	150	107	89	49	61	67	71	58	60	54	39	28
**65**–**69**	155	172	146	121	93	101	104	103	101	94	101	96	72
**70**–**74**	175	171	132	143	131	122	138	159	152	146	143	158	153
**75**–**79**	191	207	174	159	161	213	243	211	232	250	251	259	288
**80**–**84**	170	239	254	210	197	272	319	355	321	373	413	432	457
**>85**	189	294	349	437	456	465	708	910	1049	1090	1247	1473	1620
**Total number of deaths**	1330	1548	1365	1345	1213	1359	1724	1908	2005	2083	2262	2506	2673
(**C**) **Women**													
**Observed NMSC Deaths**									**Projected NMSC Deaths**				
**Age** (**Years**)	**1980**–**1984**	**1985**–**1989**	**1990**–**1994**	**1995**–**1999**	**2000**–**2004**	**2005**–**2009**	**2010**–**2014**	**2015**–**2019**	**2020**– **2024**	**2025**–**2029**	**2030**–**2034**	**2035**–**2039**	**2040**– **2044**
**0**–**4**	0	1	0	0	0	0	0	0	0	0	0	0	0
**5**–**9**	0	0	0	0	0	1	0	0	0	0	0	0	0
**10**–**14**	0	0	0	0	0	0	0	0	0	0	0	0	0
**15**–**19**	0	1	1	0	0	0	0	0	0	0	0	0	0
**20**–**24**	1	3	1	1	3	1	0	2	1	1	1	1	1
**25**–**29**	1	6	2	4	1	2	4	2	3	3	3	3	3
**30**–**34**	3	6	2	6	3	1	5	1	1	0	0	0	0
**35**–**39**	5	12	4	2	4	4	3	4	1	1	0	0	0
**40**–**44**	5	11	7	9	6	7	11	4	8	2	2	1	1
**45**–**49**	13	8	10	8	10	6	17	8	4	8	3	2	2
**50**–**54**	16	11	9	15	7	9	9	14	10	5	10	4	3
**55**–**59**	29	24	16	6	13	16	23	13	18	11	8	17	7
**60**–**64**	33	26	28	25	20	21	33	19	18	28	23	15	30
**65**–**69**	45	44	47	35	24	28	36	32	29	22	36	33	23
**70**–**74**	90	87	64	58	57	50	44	36	46	45	38	63	59
**75**–**79**	142	141	109	105	86	90	90	68	75	81	84	78	123
**80**–**84**	160	244	230	165	168	161	179	169	127	148	167	182	175
**>85**	344	540	641	691	678	681	832	1004	933	875	1004	1211	1374
**Total number of deaths**	887	1165	1171	1130	1080	1078	1286	1376	1272	1231	1378	1611	1802

NMSC: Non-melanoma Skin Cancer

**Table 2:** Age-specific mortality rates per 100,000 inhabitants (European population 2013) observed (1980–2019) and projected (2020–2044) by periods and age groups by NMSC in Spain. (**A**) Both sexes; (**B**) men; (**C**) women.

**(A) Both Sexes**													
**Age (Years)**	**1980**–**1984**	**1985**–**1989**	**1990**–**1994**	**1995**–**1999**	**2000**–**2004**	**2005**–**2009**	**2010**–**2014**	**2015**–**2019**	**2020**– **2024**	**2025**–**2029**	**2030**–**2034**	**2035**–**2039**	**2040**–**2044**
**0**–**4**	0	0.01	0	0	0	0	0	0	0	0	0	0	0
**5**–**9**	0.01	0.01	0.01	0	0	0.01	0	0	0	0	0	0	0
**10**–**14**	0.01	0	0.01	0	0	0	0	0	0	0	0	0	0
**15**–**19**	0.02	0.02	0.02	0	0.01	0.01	0	0	0	0	0	0	0
**20**–**24**	0.01	0.02	0.01	0.01	0.03	0.01	0.01	0.02	0.01	0.01	0.01	0.01	0.01
**25**–**29**	0.04	0.09	0.08	0.08	0.04	0.02	0.05	0.02	0.03	0.03	0.03	0.03	0.03
**30**–**34**	0.09	0.12	0.14	0.13	0.05	0.03	0.04	0.03	0.02	0.02	0.02	0.01	0.01
**35**–**39**	0.14	0.24	0.14	0.12	0.08	0.06	0.05	0.04	0.03	0.03	0.02	0.02	0.02
**40**–**44**	0.24	0.37	0.26	0.22	0.1	0.11	0.15	0.05	0.05	0.05	0.04	0.04	0.03
**45**–**49**	0.48	0.51	0.33	0.31	0.22	0.1	0.2	0.11	0.06	0.06	0.06	0.06	0.05
**50**–**54**	0.99	0.88	0.42	0.49	0.3	0.25	0.3	0.24	0.18	0.1	0.11	0.11	0.11
**55**–**59**	1.57	1.24	0.79	0.62	0.49	0.6	0.55	0.37	0.37	0.29	0.17	0.2	0.21
**60**–**64**	1.9	1.77	1.26	1.08	0.72	0.72	0.81	0.67	0.48	0.5	0.42	0.28	0.32
**65**–**69**	2.78	2.72	2.06	1.54	1.15	1.37	1.26	1.13	0.99	0.76	0.82	0.73	0.5
**70**–**74**	4.28	4.05	2.74	2.37	2.01	1.8	2.06	1.88	1.73	1.53	1.25	1.39	1.25
**75**–**79**	7.49	6.92	5.36	4.38	3.39	3.66	3.91	3.54	3.24	3.14	2.91	2.51	2.77
**80**–**84**	13.24	15.54	13.35	9.55	7.94	7.52	7.42	7.44	6.83	6.47	6.49	6.25	5.51
**>85**	38.18	45.57	41.12	37.05	31.87	26.16	26.55	26.87	25.27	24.46	23.98	24.57	23.87
**SMR**	2.31	2.5	2.05	1.74	1.43	1.28	1.32	1.25	1.15	1.09	1.04	1.03	0.99
**(B) Men**													
**Age** (**Years**)	**1980**–**1984**	**1985**–**1989**	**1990**–**1994**	**1995**–**1999**	**2000**–**2004**	**2005**–**2009**	**2010**–**2014**	**2015**–**2019**	**2020**– **2024**	**2025**–**2029**	**2030**–**2034**	**2035**–**2039**	**2040**–**2044**
**0**–**4**	0	0	0	0	0	0	0	0	0	0	0	0	0
**5**–**9**	0.01	0.01	0.02	0	0	0	0	0	0	0	0	0	0
**10**–**14**	0.01	0	0.01	0	0	0	0	0	0	0	0	0	0
**15**–**19**	0.04	0.02	0.02	0	0.02	0.02	0	0	0	0	0	0	0
**20**–**24**	0.01	0.01	0	0.01	0.02	0	0.02	0	0.01	0.01	0.01	0.01	0.01
**25**–**29**	0.06	0.09	0.14	0.11	0.07	0.01	0.04	0	0.02	0.02	0.02	0.02	0.02
**30**–**34**	0.13	0.15	0.26	0.18	0.06	0.05	0.03	0.05	0.04	0.04	0.03	0.03	0.03
**35**–**39**	0.19	0.29	0.21	0.21	0.11	0.08	0.07	0.03	0.06	0.05	0.04	0.04	0.04
**40**–**44**	0.38	0.55	0.41	0.3	0.13	0.14	0.18	0.05	0.04	0.07	0.06	0.06	0.05
**45**–**49**	0.75	0.87	0.49	0.49	0.3	0.13	0.22	0.14	0.08	0.05	0.09	0.08	0.08
**50**–**54**	1.73	1.59	0.66	0.73	0.49	0.38	0.49	0.33	0.26	0.15	0.1	0.18	0.18
**55**–**59**	2.67	2.11	1.33	1.14	0.77	0.97	0.78	0.58	0.53	0.43	0.27	0.2	0.34
**60**–**64**	3.28	3.18	2.1	1.77	1.07	1.1	1.12	1.1	0.77	0.71	0.61	0.41	0.32
**65**–**69**	4.96	4.83	3.39	2.57	1.96	2.28	1.97	1.82	1.65	1.32	1.27	1.13	0.8
**70**–**74**	6.73	6.45	4.31	3.82	3.13	2.83	3.4	3.32	2.91	2.58	2.17	2.14	1.93
**75**–**79**	10.92	10.47	8.46	6.56	5.3	6.06	6.6	6.1	5.62	5.46	5.02	4.42	4.37
**80**–**84**	19.08	21.34	19.46	15.04	11.66	12.32	12	12.47	11.97	11.29	11.23	10.65	9.54
**>85**	43.55	51.74	46.62	46.59	42.59	34.53	37.94	38.19	37.67	37.14	36	36.41	34.9
**SMR**	3.21	3.41	2.76	2.46	2.03	1.87	1.98	1.92	1.82	1.73	1.64	1.59	1.5
**(C) Women**													
**Age** (**Years**)	**1980**–**1984**	**1985**–**1989**	**1990**–**1994**	**1995**–**1999**	**2000**–**2004**	**2005**–**2009**	**2010**–**2014**	**2015**–**2019**	**2020**– **2024**	**2025**–**2029**	**2030**–**2034**	**2035**–**2039**	**2040**–**2044**
**0**–**4**	0	0.02	0	0	0	0	0	0	0	0	0	0	0
**5**–**9**	0	0	0	0	0	0.02	0	0	0	0	0	0	0
**10**–**14**	0	0	0	0	0	0	0	0	0	0	0	0	0
**15**–**19**	0	0.01	0.01	0	0	0	0	0	0	0	0	0	0
**20**–**24**	0.01	0.04	0.01	0.01	0.04	0.01	0	0.04	0.02	0.02	0.02	0.02	0.02
**25**–**29**	0.02	0.08	0.03	0.05	0.01	0.02	0.05	0.03	0.04	0.04	0.04	0.04	0.04
**30**–**34**	0.05	0.09	0.03	0.07	0.04	0.01	0.05	0.01	0.01	0	0	0	0
**35**–**39**	0.09	0.2	0.06	0.03	0.05	0.04	0.03	0.04	0.01	0.01	0.01	0	0
**40**–**44**	0.1	0.19	0.11	0.14	0.08	0.08	0.12	0.04	0.08	0.03	0.02	0.02	0.02
**45**–**49**	0.22	0.16	0.17	0.13	0.15	0.07	0.19	0.09	0.04	0.09	0.03	0.03	0.03
**50**–**54**	0.27	0.19	0.18	0.26	0.11	0.13	0.11	0.16	0.1	0.05	0.11	0.04	0.04
**55**–**59**	0.54	0.42	0.29	0.12	0.22	0.25	0.33	0.16	0.21	0.15	0.08	0.18	0.08
**60**–**64**	0.72	0.5	0.5	0.45	0.4	0.36	0.52	0.28	0.22	0.32	0.25	0.16	0.32
**65**–**69**	1.11	1	0.93	0.64	0.44	0.56	0.62	0.51	0.43	0.28	0.42	0.36	0.24
**70**–**74**	2.51	2.34	1.57	1.23	1.1	0.96	0.92	0.65	0.77	0.69	0.5	0.77	0.67
**75**–**79**	5.27	4.62	3.38	2.91	2.02	1.89	1.86	1.54	1.46	1.44	1.38	1.11	1.59
**80**–**84**	9.99	12.27	9.92	6.52	5.78	4.53	4.41	4.02	3.31	3.24	3.35	3.37	2.81
**>85**	35.76	42.79	38.63	32.8	27.26	22.45	21.15	21.18	17.52	15.98	16.26	17.2	17.28
**SMR**	1.67	1.86	1.57	1.28	1.06	0.89	0.88	0.81	0.69	0.63	0.63	0.65	0.65

SMR: Standardized mortality rate.

## Data Availability

The observed population data (1980–2019) and projected 82020-2044) are publicly accessible through the INE website (www.ine.es). Data on the number of deaths by age are available upon request from the corresponding author. The data is not publicly available due to restrictions in the INE’s data transfer policy.

## Supplementary Materials

The following are available online at https://www.mdpi.com/article/10.3390/jcm10245750/s1, Supplementary Material: R code.
