# Supplementary material for: Non-Melanoma Skin Cancer Mortality in Spain: A Predictive Model up to 2044
Source: J Clin Med. 2021 Dec 8;10(24):5750. doi: 10.3390/jcm10245750 (PMC8703753; doi:10.3390/jcm10245750)
Supplement: Supplementary file 1 [file jcm-10-05750-s001.zip › jcm-1466420-supplementary.pdf]

Supplementary Material-R code

```
# Reading package:
source("Nordpred.S")
options(scipen = 999)

# Reading data
indata <- read.table("data//varones_casos.txt",header =T,sep=";",row.names=1)
inpop1 <- read.table("data//varones_actual.txt",header =T,sep=";",row.names=1)
inpop2 <- read.table("data//varones_pred.txt",header =T,sep=";",row.names=1)

# Include possible population predictions
inpop <- cbind(inpop1,inpop2)

# Run predictions:
est <- nordpred.estimate(cases=indata,pyr=inpop,noperiod=5,startestage=7)
res <-
nordpred.prediction(est,startuseage=7,cuttrend=c(0,.25,.5,.75,.75),recent=T)



# Or with poisson link function (instead of the powerlink as used in the
nordpred predictions):
est2 <- nordpred.estimate(indata,inpop,5,7,linkfunc="poisson")
res2 <-
nordpred.prediction(est2,startuseage=7,cuttrend=c(0,.25,.5,.75,.75),recent=T)




# Get results:
print.nordpred(res)
nordpred.getpred(res)
summary(res,printpred=F)

# Get results with standardisation:
# World population
wstand <- c(0.12, 0.1, 0.09, 0.09, 0.08, 0.08, 0.06, 0.06, 0.06, 0.06,0.05,
            0.04, 0.04, 0.03, 0.02, 0.01, 0.005, 0.005)

European population 2013
wstand2 <- c(0.05, 0.055, 0.055, 0.055, 0.06, 0.06, 0.065, 0.07, 0.07, 0.07,
0.07, 0.065, 0.06, 0.055, 0.05, 0.04, 0.025, 0.025)




round(nordpred.getpred(res,incidence=T,standpop=NULL),2)
round(nordpred.getpred(res,incidence=T,standpop=wstand),2)
round(nordpred.getpred(res,incidence=T,standpop=wstand2),2)

# Plot results:
plot(res,standpop=wstand)
```

```
# Plot results with power5 and poisson links:
plot(res2,standpop=wstand)
plot(res,new=T,lty=c(1,2),standpop=wstand)

#plot Pob estandar europea
plot(res,standpop=wstand2)
plot(res2,standpop=wstand2)
plot(res,new=T,lty=c(1,2),standpop=wstand2)


plot(nordpred.prediction(est,startuseage=7,cuttrend=c(0,.25,.5,.75,.75),recent
=T),standpop=wstand,new=T,lty=c(1,4))
plot(nordpred.prediction(est,startuseage=7,cuttrend=c(0,.25,.5,.75,.75),recent
=T),standpop=wstand2,new=T,lty=c(1,4))
```
